# Quality of Life With Ehlers‐Danlos Syndrome/Joint Hypermobility Syndrome: A Systematic Review of Psychosocial Interventions

**DOI:** 10.1002/msc.70070

**Published:** 2025-02-15

**Authors:** Erika Bohling‐Davis, Boushra Khan‐Lodhi, Elizabeth Jenkinson, Maddie Tremblett, Jane Meyrick

**Affiliations:** ^1^ School of Social Sciences Frenchay Campus University of the West of England Bristol England

**Keywords:** Ehlers‐Danlos syndrome, joint hypermobility syndrome, psychosocial interventions, quality of life, systematic review

## Abstract

**Background:**

Psychosocial interventions may improve QoL in people with wider chronic pain conditions. However, the evidence requires refining for application to EDS/JHMS. This systematic review aimed to identify, assess and synthesise the evidence of the effectiveness of psychosocial interventions concerning EDS/JHMS. EBSCO, OpenGrey, Cochrane, Prospero, Researchgate and BPS Wiley online were searched for papers published approximately 2000–2024 for studies in which (1) Participants diagnosed with EDS/JHMS. (2) Quantitative or mixed methods. (3) Assessed a Psychosocial intervention to a (4) quality of life outcome. (5) in English. EPHPP quality assessment tool was used to assess the quality and risk of bias.

**Main text:**

The study identified six studies, including 343 participants aged 13–69 (*F* = 248, *M* = 8), of unknown ethnicity. Five studies were cohort and one non‐randomised controlled trial. Key methodological flaws included no reported effect size and no control group. With quality assessed as low (5) or moderate (1), there was weak evidence that psychosocial interventions containing mindfulness and CBT resulted in a general improvement in QoL compared to no intervention.

**Conclusions:**

Findings from this review indicate the potential of mindfulness and CBT in improving QOL in EDS/JHMS and, in some studies, pain and fatigue. However, existing research is at high risk of bias, has low methodological quality, and is predominately focused on female patients. Future research should adopt methodologically robust approaches such as RCTs and more inclusive samples and consider co‐production.

## 1 Introduction

Ehlers‐Danlos syndrome/joint hypermobility syndrome (EDS/JHMS) is a debilitating chronic health condition that significantly impacts a person’s daily living. These syndromes are a group of inherited connective tissue disorders characterised by weak and faulty collagen (Ghali, Sobey and Burrows 2019). There are multiple types of EDS; this paper will focus on the hypermobility type, sometimes referred to as joint hypermobility syndrome (JHMS) or hypermobility spectrum disorder (HSD), as it is the most common type (Ghali, Sobey and Burrows 2019). EDS/JHMS can be identified by symptoms including but not limited to fragile skin, easy bruising, chronic pain, fatigue, joint hypermobility, easy dislocations, hypertrophic scarring and rupture of blood vessels and internal organs (Ghali, Sobey and Burrows 2019). The extent to which these symptoms affect individuals varies, with some able to live an everyday life and others ultimately bed‐bound (Ghali, Sobey and Burrows 2019).

There is a lack of consensus on how to treat EDS/JHMS best. Treatment is limited in EDS/JHMS and it currently has no specific treatment protocol but has some recommended management techniques with little evidence regarding their success. Commonly used management techniques for EDS/JHMS include but are not limited to psychological therapy, pain management interventions, medication, physiotherapy and occupational therapy (Song 2020). Common medications prescribed to treat chronic pain in EDS/JHMS include Codeine, Tramadol and other opiate‐based medications (Song 2020). These drugs have a wide range of adverse side effects, such as constipation, brain fog, and addiction, in the face of decreasing effectiveness (Chopra et al. 2017). Other pain medications, such as non‐steroidal anti‐inflammatories (NSAIDs), bring their risks, such as stomach ulcers, bleeding and reflux (Chopra et al. 2017). As EDS/JHMS is a multi‐systemic condition, patients often experience symptoms such as constipation, reflux, and brain fog, which these medications may worsen and exacerbate (Ghali, Sobey and Burrows 2019). However, it is important to note that medications themselves can also improve QoL in EDS/JHMS (Gericke et al. 2024). A lack of clear treatment pathways can also impact patients' psychological health if they feel there is nothing to help improve their quality of life (QoL), and they become increasingly isolated due to decreasing physical health (Berglund et al. 2015).

It is important to consider how EDS/JHMS impacts people on a biopsychosocial level. Biologically, a person can experience extreme physical pain, fatigue, multi‐systemic organ dysfunction and frequent dislocations (B. Berglund and Nordström 2001; Castori et al. 2013). Psychologically, the difficulty of living with EDS/JHMS can increase a person's likelihood of developing psychological illnesses such as depression and anxiety (B. Berglund and Nordström 2001; Castori et al. 2013). Socially/economically, EDS/JHMS can reduce a person’s ability to socialise and work, causing loneliness, exclusion from social life and economic difficulty (B. Berglund and Nordström 2001; Castori et al. 2013). The biopsychosocial impacts of EDS/JHMS intertwine to shape how a person experiences the condition. For example, low mood (psychological) as a result of pain (biological) can increase loneliness and social isolation (social), which can impact low mood (psychological) and impact pain (biological) and the cycle continues. Because of the combination of biopsychosocial symptoms such as pain, fatigue, social isolation and economic difficulty, people with EDS/JHMS have significantly reduced QoL (Berglund et al. 2015; Rombaut et al. 2011). A recent systematic review and thematic analysis concluded that people lived a ‘restricted life’ that ultimately impacted their QoL (Bennett et al. 2019). However, who measures ‘good’ in EDS/JHMS outcomes regarding improvement in QoL is unclear, and listening to patients' experiences could help understand this (de Bienassis et al. 2022).

Because of the biopsychosocial impact on EDS/JHMS, treatment must take a biopsychosocial approach. Psychosocial interventions are a type of behavioural intervention and have both psychological and social components (Sobel 1995). They are based on the biopsychosocial approach and how biological, psychological and social factors can influence how a person experiences life and illness (Karimi and Brazier 2016). Psychosocial interventions aim to improve people's lives by improving psychological and social functioning. For example, developing coping strategies, helping motivation and improving communication techniques (Portelli 2018). Psychosocial interventions can be informal, such as general advice and education, or specific techniques, including but not limited to motivational interviewing (MI), cognitive behavioural therapy (CBT), or mindfulness (Portelli 2018). They are used frequently in healthcare, community outreach and social work and are often delivered by a multi‐disciplinary team (Berkman and Kawachi 2000). Studies, such as Meints and Edwards (2018), show psychosocial interventions may improve QoL, reduce pain and reduce the need for medication for people with chronic pain conditions. Furthermore, recently published papers emphasise the importance of treating EDS/JHMS from a biopsychosocial perspective (Baeza‐Velasco et al. 2011; Bennett et al. 2019; Clark et al. 2023).

Despite the need for psychosocial interventions due to the biopsychosocial impact of EDS/JHMS, there is a limited evidence base for their effectiveness. This lack of research is reflected in current practice and policy. For example, the NHS notes no specific treatment for EDS/JHMS but recommends physiotherapy, occupational therapy, pain management, and CBT (NHS 2017). This recommendation is mirrored in other countries (Ghali, Sobey and Burrows 2019; Mayo clinic 2024). Despite this, physiotherapy is the most frequently recommended treatment in multiple countries and the psychosocial impacts of the disease can impact a person significantly but are often overlooked (Mayo clinic 2024). Understanding the effectiveness of psychosocial interventions to improve QoL in people with EDS/JHMS could help solidify a treatment regime. Therefore, a systematic review of the literature is needed.

Treatment plans for EDS/JHMS could learn from interventions that include a psychosocial focus and are effective for other chronic conditions. For example, one systematic review and meta‐analysis of 13 studies and 1617 participants on the effectiveness of psychosocial interventions for people with multiple sclerosis found significant improvement in health‐related QoL, fatigue, anxiety and depression (Sesel, Sharpe and Naismith 2018). A further study found that psychosocial interventions, particularly CBT and mindfulness, improve QoL in people with rheumatoid arthritis (Sharpe 2016). Similarly, a recent systematic review of 11 studies and 1275 participants on the ability of psychosocial interventions to reduce pain in late‐stage cancer patients found a small but significant decrease in pain scores post‐intervention (Warth et al. 2020). Finally, another systematic review of 13 studies found that psychosocial interventions can improve social functioning in young people with physical health conditions (Forgeron et al. 2018). This is important because if psychosocial interventions improve QoL in other pain‐inducing long‐term conditions, research is needed to determine if they have similar effects in EDS/JHMS. This is because if they do, it could help improve patients' QoL.

Given the current lack of a clear treatment regime for EDS/JHMS patients and the potential for psychosocial interventions to help improve QoL, the current systematic review of the literature aimed to understand if psychosocial interventions improve QoL in people with EDS/JHMS and had the objective of narratively synthesising the current literature.

## 2 Methods

The methodology followed the PRISMA guidance (Moher et al. 2009).

### 2.1 Eligibility Criteria

Included studies had to meet the following criteria: (1) participants diagnosed with Ehlers‐Danlos syndrome, joint hypermobility syndrome or hypermobility spectrum disorder. (2) Quantitative or mixed methods design (quantitative element extracted). (3) Psychosocial intervention. (4) Assess quality of life. (5) in English. Exclusion criteria were (1) Diagnosis other than EDS/JHMS/HSD. (2) Full text unavailable. (3) No English version. These criteria aligned with the guidance of the Cochrane Handbook for Systematic Reviews (Cochrane Training 2024), which suggests that having strict eligibility criteria aids quality.

For this study, psychosocial interventions included one of the following: biopsychosocial theory, interventions including but not limited to CBT, acceptance and commitment therapy (ACT), general psychosocial intervention, motivational interviewing (MI), or mindfulness. Similarly, quality of life (QoL) assessment was defined as any scale measuring QoL and the potential building blocks, including fatigue, pain, anxiety, depression, social life and function. Research suggests that they are all significantly related to a person's QoL (Karimi and Brazier 2016). Furthermore, the National Institute for Health and Care Excellence (NICE) defines health‐related QoL as a combination of physical, psychological, and social wellbeing (NICE 2023). This means that studies that measured areas that impact QoL, such as pain, fatigue, and mood, were included, not just studies that used a specific QoL outcome measure. As such, when discussing whether studies show improvement in QoL, this systematic review refers to outcome measures that assess QoL as a broader term (such as pain, fatigue and mood), not just a specific QoL outcome measure unless specified otherwise.

### 2.2 Literature Search

The current study used guidance on selecting and extracting data from Boland et al. (2017) and searched EBSCO, Open Grey, Researchgate, Cochrane, Prospero and BPS Wiley Online databases between January 2000 and June 2023. Databases were searched using participant, intervention, comparison and outcome (PICO) to structure a well‐built research question and find relevant studies (Eriksen and Frandsen 2018). The search terms can be seen in Table 1. Grey literature was searched on Open Grey to avoid publication bias.

**TABLE 1:** Search terms.

Population	Intervention	Outcome	Boolean search terms
Ehlers Danlos syndrome, EDS, Ehlers Danlos syndrome, Hypermobility syndrome, HMS, Joint hypermobility syndrome, JHMS, Hypermobility, hypermobility spectrum disorder, HSD.	Psychosocial, Psychosocial interventions, Quantitative, Cognitive behavioural therapy, CBT, Acceptance and commitment therapy, ACT, Mindfulness, Motivational interviewing, MI.	Quality of life, QOL, Fatigue, Pain, Function, Health related quality of life, HqoL, Wellbeing.	TITLE: ABSTRACT: AND NOT ‘’

### 2.3 Study Selection and Data Extraction

**FIGURE 1:**
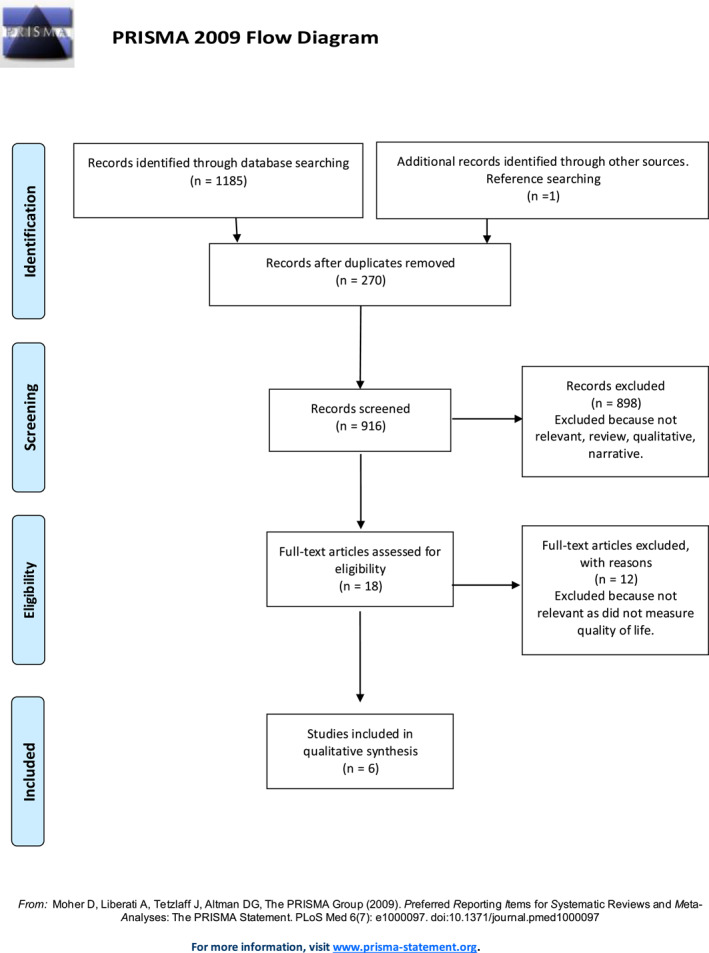
PRISMA flow diagram (Moher et al. 2009).

Databases were searched, potentially relevant articles were saved for screening, abstracts were screened against inclusion criteria, and full‐text articles were then reviewed against inclusion criteria. A PRISMA flow diagram was completed to show the search and selection process and reasons for excluding papers from the systematic review (see PRISMA flow diagram Figure 1). This process was used because it is shown to be systematic and reliable in that it follows a set and easily replicable process, which is essential for future research (Vu‐Ngoc et al. 2018). The second author repeated this process at every stage to ensure no studies were missed and the process was replicable (Cochrane Training 2024). A simplified version of the Cochrane data extraction tool for RCTs was tailored to the study selection criteria. The relevant information gathered was later combined into an Excel spreadsheet.

## 3 Results and Analysis

The current systematic review aimed to synthesise evidence from studies investigating whether psychosocial interventions improved QoL in people with EDS/JHMS. Studies identified were combined using narrative synthesis due to the clinical, statistical, and methodological heterogeneity of the included papers (Cochrane Training 2024). Results will first include a narrative of cross‐study characteristics and then discuss the evidence of intervention effectiveness.

### 3.1 Participants

Overall, there were 343 participants between 13 and 69 years of age of unknown ethnicities from France (Hakimi, Bergoin and Mucci 2020), Italy (Celletti et al. 2021), Norway (Bathen et al. 2013), and England (Kalisch et al. 2022; Lattimore and Harrison 2022; Rahman, Daniel and Grahame 2014). Four studies (Bathen et al. 2013; Celletti et al. 2021; Hakimi, Bergoin and Mucci 2020; Rahman, Daniel and Grahame 2014) were recruited from rehabilitation centres, and two (Kalisch et al. 2022; Lattimore and Harrison 2022) were conducted via opportunity sampling through social media. Four studies (Bathen et al. 2013; Celletti et al. 2021; Hakimi, Bergoin and Mucci 2020; Lattimore and Harrison 2022) had participants with similar age ranges (13–69), showing some clinical homogeneity. However, the two studies (Kalisch et al. 2022; Rahman, Daniel and Grahame 2014) do not clearly define the age range of participants. Similarly, all studies included had different participant characteristics and used different inclusion criteria regarding participant age, sex, and ethnicity. As such, overall, the studies were clinically heterogeneous. Most of the participants were female and of unknown ethnicity, meaning the results do not represent males or non‐binary people, and it is unclear what ethnicity the results represent. Interventions were either CBT or mindfulness‐based, but three included other aspects, such as physiotherapy. This means that if the interventions are effective, it is unclear if this is due to the psychosocial or physiotherapy part of the intervention. Interventions were 2–12 weeks and were run by multi‐disciplinary teams, including psychologists, nurses and physiotherapists(Table 2).

**TABLE 2:** Study characteristics.

Authors	Participants and recruitment	Study design, analysis, and outcome measures	Intervention	Results (significant if *p* *=* < 0.05)	Quality assessment
					1 = strong
					2 = moderate
					3 = weak
(Bathen et al. 2013)	N: 21	Cohort study No control Wilcoxon signed‐rank test Numeric pain rating scale (NPRS), Canadian occupational performance measure (COPM). Measured pre‐and‐post‐intervention.	12‐week cognitive behavioural and physiotherapy intervention delivered by a multi‐disciplinary team. Using a CBT approach, they aimed to increase awareness of thought patterns regarding pain and disability. Exercises focused on strength and coordination, and participants were given pacing information.	NPRS pre: 7 (4–10) post: 7 (4–9)	Selection bias: 2
	Age: 20–51			Wilcox *z* = 0.30	Study design: 2
	M: 0			*p* = 0.213	Confounders: 3
	F: 12			COPM pre: 2.95 (1.80–5.50) Post:4.08 (2.50–7.60)	Blinding: 3 Methods: 3
	City hospital rehabilitation centre.			Wilcox *z* = 2.67 *p* = 0.008	Overall: 3
	Norway.				
(Hakimi, Bergoin, and Mucci 2020)	N: 21	Cohort study No control ANOVA Multidimensional fatigue inventory (MFI = 20), brief pain inventory (BPI), quality of life medical outcome study short form 36 (SF‐36) pre and post‐intervention.	9‐week physical and psychoeducational CBT‐based intervention lasted 81 h in total. Two‐thirds of the time was spent on physical activity and one‐third was on wellbeing. The well‐being aspect included yoga, art therapy and patient education, which aimed to change thought patterns and manage problems caused by the disease.	BPI (*p* = 0.023), MFI‐20 (*p* = 0.01), SF‐36 (*p* = 0.001)	Selection bias: 3
	Age: 21–69				Study design: 2
	M: 1				Confounders: 3
	F: 20				Blinding: 2
	Retrospective cohort data from city hospital rehabilitation centre.				Methods: 2 Overall: 3
	France.				
(Rahman, Daniel, and Grahame 2014)	N: 87	Outpatient rehabilitation programme No control Wilcoxon and two‐tailed *t*‐test Pain self‐efficacy score (PSEQ), pain catastrophising scale, depression, anxiety and positive outlook scale (DAPOS) and brief pain inventory (BPI) pre‐and‐post intervention.	6‐week (42 h total) intervention led by physiotherapists, nurses, and psychologists and used CBT. Patients set individual goals they work towards. One and 5‐month follow‐up.	Five‐month follow‐up: Two‐tailed *t*‐tests: PSEQ (*p* = 0.002), BPI impact on daily life (*p* = 0.001), BPI average pain intensity (*p* = 0.138) Wilcoxon PCS (*p* = 0.001), DAPOS anxiety (*p* = 0.013), DAPOS depression (*p* = 0.015) 5‐month follow‐up.	Selection bias: 2
	Age: Mean 35				Study design: 3
	M: 4				Confounders: 3
	F: 83				Blinding: 3
	Rehabilitation cohort data.				Methods: 2
	England.				Overall: 3
(Celletti et al. 2021)	*N* = 18	Non‐randomised clinical trial No control Wilcoxon test for paired samples McGill pain questionnaire, fatigue severity scale (FSS), Oswestry disability index (ODI), Tampa scale (TSK) and numerical pain rating scale (NPRS) pre‐and post‐intervention.	10‐week neurocognitive rehabilitation intervention with mindfulness qualities. 60 min per week. Focused on the felt sense (how participants perceive bodily sensations) guided by physiotherapists.	Significant reduction in pain symptoms (McGill *p* = 0.03) NPRS (*p* = 0.003), fatigue (FFS *p* = 0.03), and pain related disability (owestry disability index *p* = 0.03)	Selection bias: 2
	Age: 13–55				Study design: 2
	M: 4				Confounders: 3
	F: 14				Blinding: 3
	City hospital rehabilitation centre.				Methods: 3 Overall: 3
	Italy.				
(Lattimore and Harrison 2022)	N: 76	Cohort study No control Wilcoxon ranked test QoL SF (36) pre and post‐intervention	2‐week online mindfulness intervention. Participants receive an instructional video on how to perform guided meditation. Guided meditations are focused on breathing and last 6 min. They focus on ‘noticing’, ‘gathering attention’ and ‘expanding’.	SF (36) (*p* = 0.046)	Selection bias: 3 Study design: 2 Confounders: 3 Blinding: 3 Methods: 1 Overall: 3
	Age: 26–51				
	M: 1				
	F: 75				
	83% caucasian				
	Opportunity sampling (social media).				
	England				
(Kalisch et al. 2022)	N: 132	Cohort study Waitlist control group and intervention group MANOVA and Tukey post‐hoc Discreet visual fatigue rating scale, seven‐item pain disability index (PDI), positive and negative affect scale (PANAS), and five‐item satisfaction with life scale (SWLS) pre‐ and post‐intervention.	5‐week online positive psychology intervention. Approximately 60 min a week. Activities focused on: Using strengths, planning socialising, self‐reflection, compassion, practising savouring, mindful observation, planning kindness days, goal setting, creative writing and daily reflection on three positive things. This combined mindfulness and CBT theory regarding paying non‐judgemental attention to the body and changing thought patterns.	The waitlist control group had statistically significant differences. Compared to the intervention group, the control group reported significantly lower satisfaction with life (*p* = 0.033), positive affect (*p* = 0.014) and fatigue scores (*p* = 0.028).	Selection bias: 2
	Age: Mean 37.7				Study design: 2
	M: 5				Confounders: 3
	F: 127				Blinding: 3
	Opportunity sampling (social media and newsletters)				Methods: 3 Overall: 3
	England				

### 3.2 Quality Assessment

Quality assessment enables researchers to assess whether the results of the included papers are robust and, therefore, if conclusions of significance are justified (Wells and Littell 2009). It will be used in the reporting and synthesis to aid in discussing the robustness of the results. The current study used an adapted version of the Effective Public Healthcare Panacea Project (EPHPP) quality assessment tool for quantitative studies (EPHPP 2024). EPHPP is reliable at assessing the quality of quantitative research across multiple areas and has excellent inter‐rater reliability for the final grade (Armijo‐Olivo et al. 2012). The EPHPP assesses quality across the following areas: selection bias, study design, confounders, blinding, withdrawals and drop‐outs, intervention integrity, and analysis (EPHPP 2024). These areas are rated from 1 to 3, and scores are collated to produce an overall score for each paper, with 1 being strong, 2 being moderate and 3 being weak. Five studies had a quality assessment score of ‘weak’ and one of ‘moderate’. The primary researcher completed quality assessment forms, which a second reader then repeated to ensure the tool was reliable and the results were representative of the selected papers.

### 3.3 Study Design

Five studies used cohort data (Bathen et al. 2013; Hakimi, Bergoin and Mucci 2020; Kalisch et al. 2022; Lattimore and Harrison 2022; Rahman, Daniel and Grahame 2014), and one study was a non‐randomised clinical trial (Celletti et al. 2021). All studies collected data through surveys but used differing statistical analysis methods. Outcome measures used were; Numeric Pain Rating Scale (NPRS), Canadian Occupational Performance Measure (COPM), Multidimensional fatigue inventory (MFI = 20), Brief Pain Inventory (BPI), Quality of Life Short Form 36 (QoL SF‐36), Pain Self‐Efficacy Score (PSEQ), Pain Catastrophising Scale, Depression, Anxiety, and Positive Outlook Scale (DAPOS), Quality of Life Short Form 36 (QoL SF 36), McGill Pain Questionnaire, Fatigue Severity Scale (FSS), Oswestry Disability Index (ODI), Tampa Scale (TSK), Discreet Visual Fatigue Rating Scale (DVFRS), seven‐item Pain Disability Index (PDI), Positive and Negative Affect Scale (PANAS) and five‐item Satisfaction With Life Scale (SWLS).

All papers used outcome measures to assess aspects relating to QOL, including fatigue, pain, function, anxiety, depression, and overall QOL questionnaires. However, all studies used a different combination of these. Furthermore, all studies used different statistical analysis methods to analyse data. As such, the papers included in this systematic review are methodologically and statistically heterogeneous. This means that the results of the six included studies cannot be combined in a meta‐analysis.

### 3.4 Evidence of Intervention Effectiveness

This narrative synthesis will first discuss the CBT‐based psychosocial interventions (Bathen et al. 2013; Hakimi, Bergoin and Mucci 2020; Kalisch et al. 2022; Rahman, Daniel and Grahame 2014) and then the mindfulness‐based psychosocial interventions (Celletti et al. 2021; Kalisch et al. 2022; Lattimore and Harrison 2022), making note of any differences in results between online and face‐to‐face interventions.

#### 3.4.1 CBT Interventions

Four studies used CBT‐based interventions (Bathen et al. 2013; Hakimi, Bergoin and Mucci 2020; Kalisch et al. 2022; Rahman, Daniel and Grahame 2014). Three studies combined CBT and physiotherapy approaches utilising physical therapy and CBT exercises such as goal setting, identifying strengths, changing mindsets, and education. One study was just CBT‐based. All four found a statistically significant improvement in outcome measures relating to QoL. It is unclear in the four studies that included physiotherapy if the significant increase was due to physiotherapy, CBT, or both. All studies had weak quality assessment ratings, meaning that the results may not be reliable. Three of the CBT‐based studies were face‐to‐face (Bathen et al. 2013; Hakimi, Bergoin and Mucci 2020; Rahman, Daniel and Grahame 2014), and one was online (Kalisch et al. 2022), but all four still found statistically significant improvement in outcome measures relating to QoL.

#### 3.4.2 Mindfulness Interventions

Two studies used mindfulness‐based psychosocial interventions (Celletti et al. 2021; Lattimore and Harrison 2022). The face‐to‐face intervention (Celletti et al. 2021) was a combination of mindfulness led by physiotherapists but did not include physiotherapy. The online intervention (Lattimore and Harrison 2022) was just guided mindfulness meditation. Both found a statistically significant increase in outcome measures relating to QoL. This suggests that mindfulness‐based psychosocial interventions were both effective, whether online or face‐to‐face. However, both studies scored low on quality assessment, meaning the results may not be reliable(Table 3).

**TABLE 3:** Results overview.

Study	Statistically significant decrease in pain	Statistically significant decrease in fatigue	Statistically significant increase in QoL/anxiety and depression
Bathen et al. 2013	N	N	N/A
Hakimi, Bergoin and Mucci 2020	Y	Y	Y
Rahman, Daniel and Grahame 2014	Y	N	Y
Celletti et al. 2021	Y	Y	N/A
Lattimore and Harrison 2022	Y	N	Y
Kalisch et al. 2022	N	Y	N

Overall, the six studies showed a statistically significant improvement in outcome measures relating to QoL but were all methodologically weak due to a lack of diversity in participants and the use of cohort studies. Although there are strengths to cohort data, such as the ability to gather large amounts of multiple outcome measure data from one group, there are limitations. It is hard to account for all extraneous variables, meaning it is difficult to say whether effects are due to the intervention or something else the participants have come into contact with (Grimes and Schulz 2002). Similarly, it can be difficult to maintain contact with participants, in turn impacting researchers' ability to follow up and collect further data, which could impact the validity of the study and introduce bias (Grimes and Schulz 2002).

Furthermore, effect sizes are not reported; this is important because the smaller the effect, the weaker the relationship between variables (Sullivan and Feinn 2012). Even with a statistically significant result, if the effect size is small (e.g. *d* = 0.2 or under), the difference between the control and intervention groups is negligible (Cohen 1992). This is because statistical significance can be influenced by participant number, and with a small effect size, the study has little practical significance, meaning the effect is too small to be meaningful in clinical and daily life settings (Sullivan and Feinn 2012). Furthermore, despite the studies finding that psychosocial interventions improve the QoL in people with EDS/JHMS, the papers were of low quality, meaning the results may not be reliable.

## 4 Discussion

There is little research regarding the efficacy of psychosocial interventions for improving QoL in people with EDS/JHMS. This lack of research is reflected in current practice and policy, where the NHS notes there is no specific treatment regime for EDS/JHMS but recommends physiotherapy, occupational therapy, pain management, and CBT (NHS 2017). The current study examined whether psychosocial interventions improve quality of life (QoL) in people with EDS/JHMS through a systematic literature review.

Overall, this systematic review found an evidence gap in the literature regarding the effectiveness of psychosocial interventions in improving QoL in EDS/JHMS. This could reflect how the treatment is typically physiotherapy orientated, and the psychosocial effects of EDS/JHMS are often ignored, meaning that research primarily focuses on physiotherapy and biological therapies (Mayo clinic 2024). This is highlighted by the fact that this systematic review only found six studies across multiple databases that met the inclusion criteria. Despite this, the literature is clear that due to the biopsychosocial impact of EDS/JHMS, interventions that take a broader and more holistic approach, such as psychosocial interventions, are needed (Baeza‐Velasco et al. 2011; Bennett et al. 2019; Clark et al. 2023). This evidence gap could be due to a lack of funding and resources due to a general focus on biomedical approaches to research and the rarity of EDS/JHMS, meaning it is not seen as a priority condition (Charlton and Rid 2019; Jones and Wilsdon 2018).

Furthermore, this systematic review found inconsistencies across findings in the included studies, with some seeing a statistically significant improvement in some outcome measures and others not. Similarly, there was no consistency in who delivered the intervention, where it was delivered, and how it was delivered. This could have been impacted by how all the psychosocial interventions were different. This reflects current practice globally. For example, treatment to reduce pain and improve QoL in people with EDS/JHMS in the UK is most commonly offered at pain self‐management programs delivered by physiotherapists, psychologists, or nurses (British Pain Society 2019). These programs have guidelines set out by the British Pain Society (British Pain Society 2019) that state they should include elements such as CBT approaches, education, and physical exercise. However, there is no specific standardisation that pain management programs must follow. This means they can include third‐wave therapies such as mindfulness, acceptance and commitment therapy and other psychosocial intervention tools. This is important because a lack of standardisation in treatment regimes can impact the quality of care individuals receive. Furthermore, commonly used tools such as mindfulness are hard to define and therefore standardise (Rau and Williams 2016), further impacting the lack of standardisation of psychosocial pain management interventions. This non‐standardisation of psychosocial interventions is further represented in the literature. For example, research suggests that no specific type of psychosocial intervention is used for pain management delivered by healthcare professionals in the UK (Williams 2019). Without standardised treatment and outcome measures, there is no evidence base, meaning healthcare professionals are taking a ‘best guess’ at what works.

Furthermore, this systematic review found variability in the outcome measures, with some studies using a specific QoL outcome measure. In contrast, others used outcome measures that assessed the building blocks of QoL, such as pain, fatigue, and mood. This variability in outcome measures is important as it may not reflect patient perceptions. To further understand what elements are important to patients regarding their QoL, further research using co‐production is needed to gather patient experience and opinion so that these points can be patient‐led and guided by lived experience. The combination of non‐standardised treatment and differing outcome measures highlights the issue of who chooses what to focus on and what counts as ‘good’ regarding outcomes ‐patients or healthcare professionals. To ensure interventions are of maximum effect, these definitions must be patient‐led and guided by lived experience (de Bienassis et al. 2022).

Despite highlighting a gap in the literature, this systematic review found some promising results. For example, the studies found that psychosocial interventions that included CBT or mindfulness statistically significantly improved QoL‐related outcome measures, including pain, fatigue, and mood. Therefore, there is weak evidence that psychosocial interventions, including CBT and mindfulness, improve QoL in people with EDS/JHMS by reducing pain, reducing fatigue, improving function, or improving QoL. The evidence included in this paper is weak for common reasons such as low quality, the use of cohort designs, and lack of effect sizes. Furthermore, this paper was able to highlight gaps in the evidence base and recommend further research. More rigorous research is needed that focuses on the effectiveness of specific parts of psychosocial interventions, such as mindfulness and education, for improving QoL in people with EDS/JHMS. The design of these studies must separate out active ingredients and tie down attribution via controls to ensure that is what is causing improvement.

## 5 Future Recommendations

Due to the findings of this systematic review, the current study has the following future recommendations. Firstly, further research using randomised controlled trials (RCT) is completed to assess the effectiveness of specific aspects of psychosocial interventions in improving QoL in people with EDS/JHMS. This is because RCTs are gold standard methods to determine cause and effect, provided the sample size is large enough and representative of the parent population (Barton 2000). Secondly, qualitative investigations considering patient experience alongside robust RCTs could strengthen the evidence base by understanding from a patient perspective if psychosocial interventions do or do not improve QoL in the EDS/JHMS population. There is a focus on the medical framing of what a patient needs (i.e. medication, physiotherapy) versus what a patient wants interventions to focus on. Understanding what people with EDS/JHMS want in interventions and what is important to them regarding QoL would make them more relevant and appropriate and improve adherence to interventions. It is also important to understand if and why psychosocial interventions do or do not improve QoL in people with EDS/JHMS, which qualitative research could offer by exploring people's lived experiences (Cypress 2015). If these interventions are appropriate and effective, they could be feasible due to the cost‐effectiveness of psychosocial interventions within healthcare settings (Williams 2019). Lastly, this study recommends regularly reviewing psychosocial intervention programs for people with EDS/JHMS to ensure they meet the client's needs. This is important as it will help services refine their interventions to be the most effective for their target population.

## 6 Conclusion

In conclusion, there was weak evidence in the six included studies that psychosocial interventions improve QoL in people with EDS/JHMS. The current systematic review found that all studies showed a statistically significant increase in QoL‐related outcome measures in people with EDS/JHMS due to psychosocial interventions. However, further research is recommended due to low quality, heterogeneity of studies, potential bias, lack of effect sizes and difficulties in standardising interventions.

## Author Contributions

Mrs Erika Bohling‐Davis is the senior author, having conceptualised the idea, completed the initial analysis, written the original draft, and made final edits. Miss Boushra Khan‐Lodhi completed the secondary analysis. Dr Elizabeth Jenkinson contributed significant editing to the manuscript. Dr Maddie Tremblett contributed significant editing to the manuscript. Dr Jayne Meyrick supervised the project and commented on and reviewed the manuscript.

## Conflicts of Interest

The authors declare no conflicts of interest.

## Data Availability

Data sharing not applicable to this article as no datasets were generated or analysed during the current study.
